# A Sequential Handwriting Recognition Model Based on a Dynamically Configurable CRNN

**DOI:** 10.3390/s21217306

**Published:** 2021-11-02

**Authors:** Ahmed AL-Saffar, Suryanti Awang, Wafaa AL-Saiagh, Ahmed Salih AL-Khaleefa, Saad Adnan Abed

**Affiliations:** 1 Faculty of Computing, Universiti Malaysia Pahang (UMP), Gambang 26600, Pahang, Malaysia; ahmed_saffar5@siswa.ukm.edu.my; 2 Centre for Data Science and Artificial Intelligence (Data Science Centre), Universiti Malaysia Pahang, Kuantan 26300, Pahang, Malaysia; 3 Faculty of Information Science and Technology, Universiti Kebangsaan Malaysia UKM, Bangi 43600, Selangor, Malaysia; wafaa.alsaiagh@siswa.ukm.edu.my; 4 Department of Computer Engineering, Faculty of Information Technology, Imam Jafar Al-Sadiq University, Tehran 10011, Iraq; ahmed.salih89@siswa.ukm.edu.my; 5 Computer and Information Sciences Department, Universiti Teknologi PETRONAS, Seri Iskandar 32610, Perak, Malaysia; saadadn@gmail.com

**Keywords:** handwriting recognition, Neural Architecture Search (NAS), configuration search, metaheuristics optimization, deep learning

## Abstract

Handwriting recognition refers to recognizing a handwritten input that includes character(s) or digit(s) based on an image. Because most applications of handwriting recognition in real life contain sequential text in various languages, there is a need to develop a dynamic handwriting recognition system. Inspired by the neuroevolutionary technique, this paper proposes a Dynamically Configurable Convolutional Recurrent Neural Network (DC-CRNN) for the handwriting recognition sequence modeling task. The proposed DC-CRNN is based on the Salp Swarm Optimization Algorithm (SSA), which generates the optimal structure and hyperparameters for Convolutional Recurrent Neural Networks (CRNNs). In addition, we investigate two types of encoding techniques used to translate the output of optimization to a CRNN recognizer. Finally, we proposed a novel hybridized SSA with Late Acceptance Hill-Climbing (LAHC) to improve the exploitation process. We conducted our experiments on two well-known datasets, IAM and IFN/ENIT, which include both the Arabic and English languages. The experimental results have shown that LAHC significantly improves the SSA search process. Therefore, the proposed DC-CRNN outperforms the handcrafted CRNN methods.

## 1. Introduction

Handwritten text recognition was one of the first problems that artificial intelligence attempted to solve. Accurate recognition of handwritten text has remained a prime problem of interest for many decades. Handwriting recognition is an important task because of its ubiquity in real life in which people communicate and interact [1].

In this context, many systems and mobile applications have been developed to perceive and comprehend their visual surroundings using handwritten text recognition techniques [2,3,4,5]. The goal of these systems is to read street signs and allow easier automated navigation [6,7], search and index a large number of images or videos on the Internet [8,9,10], detect product labels for autonomous stores [11] or help in real-time text recognition of translations on smartphones [12]. These examples utilize machine vision systems that rely on various machine learning algorithms. In particular, these systems focus on deep learning techniques due to their rapid development [13].

The first attempt at a text recognition system was intended to recognize an image that includes an individual character. Recognition of the sequence of characters was achieved after the rapid growth of computer ability and the development of machine learning algorithms. The difference between character and string handwritten recognition (normal or sequential classification) can be mathematically represented.

For a handwritten character classification problem, an input *x* is an image of a character that needs to be labeled by the corresponding text character as an output called *y*. In other words, let *x* be an image of a handwritten character; then, its corresponding class is *y* ∈ A,B,C…Z. A training example set S consists of a number of samples, n and each sample is a pair of (*x*,*y*). The training set S is assumed to have been drawn from the joint distribution *P*(*x*,*y*). The intended task is to use the training set *S* to train the classifier *h* that predicts the class *y* = *h*(*x*) of the new image x as accurately as possible.

Unlike the normal supervised learning framework, some of the real-world practical applications do not fit the drawn image from the joint distribution *P*(*x*,*y*) because training data are sequential and consist of a sequence of pairs of (*x*,*y*). The handwritten sequential classification problem can be formulated as follows:

The sequential dataset S consists of a number of samples n and $S=\left(X,Y\right)$. where $X={\left(x^{\langle i\rangle \langle t\rangle }\right)}^{*}$ is the sequential input and $T_{x}\;\mathrm{is}\;\mathrm{the}\;\mathrm{timestep}$, $i\in 1,2,3,\ldots n,T_{x}=1,2,3,\ldots t$ so $x^{\langle 1\rangle \langle t\rangle }=x^{\langle 1\rangle \langle 1\rangle },x^{\langle 1\rangle \langle 2\rangle },x^{\langle 1\rangle \langle 3\rangle }\ldots x^{\langle 1\rangle \langle t\rangle }$. The input dimension $X={\left({\mathbb{R}}^{T}\right)}^{*}$ is the set of all strings of length t  of real valued vectors and their corresponding labels $Y={\left(g\right)}^{*}$. These labels are all sequences generated using a language G and its alphabet characters g. For our problem domain, we considered g elements as the timestep, or the number of distinct points, for all input sequences. The output target $Y={\left(y^{\langle i\rangle \langle t\rangle }\right)}^{*}$ is often the same length as the input sequence $X={\left(x^{\langle i\rangle \langle t\rangle }\right)}^{*}$; thus, the output target length $T_{y}=1,2,3,\ldots t\leq$ $T_{x}=1,2,3,\ldots t$. The intended task is to use the training set *S* to train the classifier h:X→Y that predicts the sequence labeling of the new image *x* as accurately as possible.

Sequential classification is typically addressed using segmentation techniques. These techniques segment string images into small pieces that correspond to individual characters or parts of characters; these characters are then recognized as sequenced text. Thereafter, the recognition results are combined with the path-search algorithms to derive the optimized global results. These techniques utilize the over-segmentation strategy, but they also face many practical issues, such as differing handwritten styles, background noise or connected characters [14]. An alternative for the handwritten text recognition task makes use of segmentation-free processes [15,16,17,18,19]. These techniques became standard methods for handwritten text recognition and rely on deep learning algorithms. In particular, these algorithms combine convolutional neural networks (CNNs) and recurrent neural networks (RNNs) [20,21], which are known as convolutional recurrent neural networks (CRNNs) [22].

Recent CRNN-based studies use complex architectures and demonstrate improved performance [23,24]. A large dataset and powerful computational infrastructure help researchers to effectively train deeper, larger and increasingly complicated architectures. However, a neural network’s performance cannot be improved by simply adding parameters or layers, as it requires the incorporation of ideas such as including skip connections [23] or creating more branches [25]. Furthermore, even popular processes such as dropout [26] and batch normalization [27] cannot always improve the system performance and must be used carefully. In contrast, architectural hyperparameters can considerably change the system performance. Thus, human experts who use a trial-and-error manual search method based on intuition closely supervise these factors.

An expert must make many choices when designing deep model specifications. These choices interact with one another in a discrete manner and can affect the system’s performance. A typical workflow helps the expert define and train a single model and thereafter compute a validation score. Depending on earlier experiences, validation scores and data collected during training, human experts must decide whether a trained model’s performance is satisfactory. When a model’s performance is found to be unsatisfactory, experts then search for variations that may improve the performance. Based on the expert’s perspective, it is more convenient to search for architecture modifications automatically, such as simple scalar hyperparameters, e.g., the regularization coefficient and learning rate. In recent years, many existing studies have applied neuroevolutionary mechanisms for automatically detecting the appropriate CNN topology and hyperparameters based on the target dataset [28,29,30,31]. Additionally, the existing automation-based CNN models have been used for character recognition.

However, there is no analytical process that can automatically search for the optimum CRNN topology and hyperparameters for handwritten text recognition. The main contribution of this paper is our proposal of a novel approach that identifies an optimal structure and hyperparameter set for the CRNN model using metaheuristic optimization techniques. Our optimized CRNN is employed for handwriting recognition at a sequence level (word level). In addition, the Salp Swarm Algorithm (SSA) is developed to optimize the CRNN. The SSA is hybridized with Late Acceptance Hill-Climbing (LAHC) as a local search algorithm that improves the SSA exploitation process. Additionally, we implemented a neuroevolutionary CRNN based on the genetic algorithm for comparison purposes. Furthermore, we investigated two solution encoding techniques for the genetic algorithm. The technique with the best performance is then implemented with the hybridized SSA.

This paper is organized into five sections, including the current section, which introduced the proposed study. Section 2 discusses related work in the field of handwriting recognition. Section 3 describes this study’s proposed method. Section 4 presents the results of our method and compares it to previous works. Finally, Section 5 draws conclusions and provides final remarks and future directions.

## 2. Literature Review

This section presents a review of works that concern the handwriting recognition problem. Specifically, this includes works that tackle this problem using deep learning algorithms (CNNs). Additionally, we review methods that studied CNN optimization, known as neuroevolutionary methods.

### 2.1. Handwriting Recognition Based on Deep Learning

Ref [32] proposed a CNN-based deep feature embedding learning scheme for flexible word detection and recognition from images comprising handwritten words. The authors emphasized that reduced feature depiction could increase convolutional features’ discerning capability and allow scaling feasibility for large datasets. Furthermore, they followed a lexicon-specific scheme that was applied to the IAM dataset. The IAM test set was used to select lexicons, and the corresponding words were used for transcription. Ref. [33] suggested implementing OCR using an integrated approach combining long short-term memory (LSTM) and convolutional neural networks. The authors used the proposed technique on challenging datasets comprising handwritten images in several languages. Additionally, they used a weighted finite-state transducer (WFST) combined with a neural model to decode complicated sets that are difficult to process using statistics-based language frameworks. The CRNN framework’s training process does not include WFST training. Original language text is employed for training CRNNs individually. The WFST statistical language framework enhances recognition execution and has a more robust decoding capability. The authors used several preprocessing steps: initially, line segmentation was executed using horizontal and vertical projections and heuristics to yield words. Subsequently, word shear was executed at the angle that provides an optimal vertical projection of histogram peaks; several angles were evaluated, and the optimal angle was selected. A straightforward slant image-fixing process was applied, and then the data were input to the network.

Ref. [34] indicates that by applying CNNs to initially compute the number of symbols composing a word block, it is possible to resize the blocks into a canonical form suitable for an FCN structure. An FCN can use the average symbol width to execute precision symbol determination without postprocessing the data using connectionist temporal classification (CTC). The model was pretrained using data from NIST and RIMES. Probabilistic CER adjustment was performed and followed by the post-correction process, which was based on a correction-specific lexicon derived from probabilistic CER. Ref. [35] implemented word detection and recognition using a combination of Bidirectional Long Short-Term Memory (BLSTM), the CTC structure, and the Spatial Transformer Network (STN). To improve recognition performance, the authors conducted pretraining with synthetic data; additionally, an STN layer was added to the CRNN model along with a language model-based lexicon for postprocessing.

Additionally, the authors suggested using a CNN-RNN combination with prepared data and domain-based image augmentation and normalization [36]. Researchers have also determined module-specific recognition improvement at the word and line levels. Ref. [37] used transfer learning (TL) on several databases with relatively fewer parameters but better generalization ability. The authors reported that a CRNN framework trained extensively using large datasets provides excellent generalization results for small historical datasets. The IAM database [38] was used for network training; subsequently, 350 lines of text were used for executing handwritten text recognition specific to the Parzival database [39]. Using a binary version of these databases, the authors reduced the test character error rate (CER) to 3.3%.

In [40], the researcher studied the impact of applying an enhanced postprocessing phase to the sequence-to-sequence (seq-to-seq) model to improve handwritten word recognition. Postprocessing consists of integrating an external language model into a sequence-to-sequence model for handwritten word recognition. The language model scheme, designated candidate fusion, was trained and aligned using the optical recognizer to prevent corpus-specific training bias; in addition, this language framework regulates decoding to achieve the most probable character set. Ref. [41] proposed an offline technique for recognizing full handwritten documents. This technique comprises a localized (text localization) handwritten text pipeline. Images of words are transformed into strings using text recognition. The CRNN framework comprises multiple CNNs used to extract image features that serve as input to a bidirectional LSTM. The preprocessing phase comprised data enrichment using shearing, random translation, and occlusions. The researchers formulated a Language Denoiser Network (LDN) for postprocessing. This network uses character-specific noise encoding and then produces clear output (words) to ensure that only in-vocabulary words are present. 

### 2.2. Neuroevolutionary Methods Based on Sequence-Less Datasets

“Neuroevolution” was an area of emphasis during the late 1980s. Its objective was to use evolutionary computation to optimize neural networks. EPNet [42] is a popular word in neuroevolution; others include neuroevolution of augmenting topologies (NEAT) [43] and EANT [44]. Neuroevolution of augmenting topologies (NEAT) is a technique that provides both neural network topology and weight to evolve several cases. Research in this area emphasized shallow neural network learning frameworks and weight regulation. The neuroevolution of deep learning can be categorized based on the encoding method used to generate a customized neural network from the given solution representation [45]. Typically, the encoding method used in deep learning neuroevolution can be divided into two categories: direct encoding and indirect encoding. For models with direct encoding, genotype representation can be directly used to map phenotypes [46,47,48]. Indirect encodings require a translatable genotype (solution decision variables) to map phenotypes (neural networks) [43,49,50,51,52]. The authors of [53] used an augmented particle swarm optimization (PSO) method to efficiently progress through block-specific CNN search. Weight-sharing algorithms were used to address the immense resources and time cost of assessing the fitness function. However, encoding addresses simple hierarchical associations. Skip connections are not accounted for and may be useful for handling complex image datasets. Similarly, encoding techniques consider the usage of only global optimization PSO without enhancing the local search procedure.

Additionally, [54] proposed an evolving CNN based on enhanced PSO called quantum behaved particle swarm optimization with binary encoding (QBPSO). However, the suggested binary encoding scheme was not yet suitable for more sophisticated CNN structures, including skip connections. Similarly, [55] discussed two techniques for melanoma classification. The first technique used HLPSO and ensemble learning-based feature identification for their lesion categorization scheme. The second categorization scheme assumed that varying CNN architectures possess the required topology and hyperparameters. Although the hybrid clustering model and deep network exhibited higher competence on two skin lesion benchmark datasets, the evolved hybrid learning PSO (HLPSO)-based CNN model must verify its stabilities by applying more common image processing benchmark datasets.

Handwriting recognition for sequence-less datasets (classification of individual characters or digits) is a common standard benchmark for several neuroevolutionary works. Ref. [56] constructed a model that tuned the CNN parameters using an optimization algorithm inspired music called the harmony search algorithm. The framework of the CNN-based harmony search algorithm aims to enhance the handwriting recognition task. Khalifa et al. [57] proposed a fixed CNN architecture for handwritten digit recognition tasks. Additionally, the PSO technique was employed to enhance the final layer weights, leading to better accuracy than most CNNs. Ref. [52] suggested a technique that could manage several aspects of CNNs, such as recurrent layers, fully connected layers, convolutional aspects, hyperparameters, and activation functions. The hyperparameters optimized in the fully connected layers are layer-based (recurrent or feed-forward). Feed-forward was selected as the optimal layer. The authors used a grammatical evolution algorithm to evolve the CNN topology. Although the framework obtained a result compatible with MNIST, a handwritten digit recognition dataset benchmark, the model did not consider using sequence time step datasets, such as handwritten words or sentences, instead of simple classification tasks. However, the use of RNNs was inefficient compared to the use of a regular feed-forward layer in the final layer.

Furthermore, related works that aim to evolve recurrent units without the use of convolutional neural networks have recently been published. Examples of these works include [58,59,60]. Ref. [60] optimized the number LSTMs by evolving its units to find the appropriate number of LSTM units for a given task. However, the work in [59] evolved only the LSTM memory cell structures themselves. Similarly, ref. [58] worked on differentiating recurrent neural networks (RNNs) by employing memory architectures such as the GRU, LSTM, the ∆-RNN, the UGRNN, and the MGU. However, such augmentations led to high complexity for large LSTM-RNN structures. Other researchers [61] focused on chaotic time-series questions and compared two decomposition techniques for training RNNs and feed-forward networks. Furthermore, connection identification pertaining to LSTM RNNs and RNNs has been attempted using the ant colony optimization (ACO) technique [62,63]. Nevertheless, this technique is constrained by a fixed RNN structure; it lacks evolvability.

Most recent studies focused on evolving convolutional neural networks (CNN) and tested them using a simple classification problem for sequence-less datasets, such as recognition of individual handwritten digits or characters. Refs. [52,56,57]. The main deferent in our work is that we tackle more challenging issues including sequence learning to recognize handwritten words or sentences. This problem needs to utilize evolving adapted deep learning algorithms which combine the benefits of the capabilities of convolutional neural networks (CNN) algorithms along with the capabilities of recurrent neural networks (RNN). The adapted deep learning algorithm (CRNN) that we evolved using the optimizing technique applied on the sequential text in various languages (Arabic and English). Additionally, in the aforementioned studies, evolving of RNN units was utilized separately without the use of any convolutional neural network. In addition, most studies used the PSO and ACO algorithms rather than investigating new swarm intelligence techniques. Additionally, previous works that studied CNN optimization did not address their imbalanced search.

## 3. Swarm Evolving-Based Automatically Configured CRNN

In this study, we propose a CRNN that addresses the problem of sequential classification. Based on the related works, the performance of a deep NN is highly dependent on its structure and hyperparameters. In this work, we focus on these two aspects of the proposed CRNN. These aspects are formulated as an optimization problem. Then, we propose a swarm intelligence algorithm known as the SSA, which is improved to solve the optimization problem. The SSA is based on multiple solutions, and thus, it emphasizes exploring the problem space rather than exploiting it. Hence, the SSA exploitation procedure has been consolidated to balance these two features. This outcome was achieved by incorporating a local search algorithm. In this study, we investigated two algorithms for local searching: Simulated Annealing (SA) and the Late Acceptance Hill-Climbing (LAHC). 

**Figure 1:**
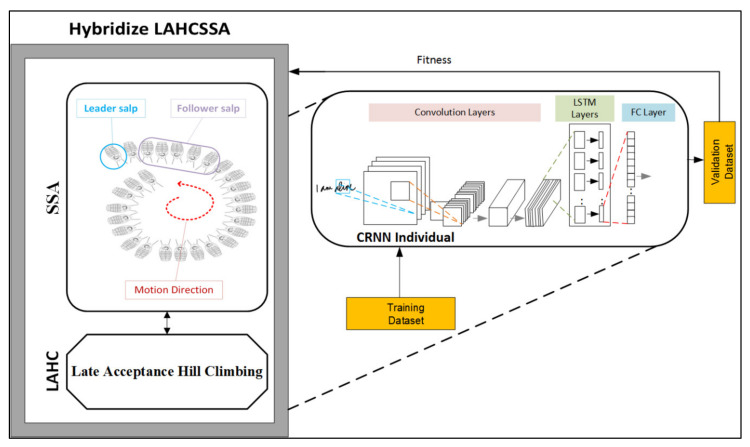
The proposed DC-CRNN framework for handwritten sequential classification.

This section introduces our proposed framework, which is capable of optimizing the hyperparameters and configuration structure of a deep learning model. Our model is called the Dynamically Configurable Convolutional Recurrent Neural Network (DC-CRNN). In this model, we used a CRNN that is dynamically configured by the proposed hybrid Salp Swarm algorithm. The framework of our DC-CRNN is presented in Figure 1.

In Figure 1, the core algorithm for handwritten sequential classification is the CRNN. Due to the sensitivity of the CRNN to its own structure and hyperparameters, this study incorporates a swarm optimization algorithm called the salp swarm algorithm (SSA). This algorithm is used to optimize the CRNN and finds the best hyperparameters and structure. The SAA emphasizes exploring the CRNN optimization space problem space rather than exploiting it. To achieve a balanced searching algorithm, we hybridize the SSA with a local search algorithm, called late acceptance hill climbing (LAHC). The local search is applied to the leader salp (encircled in blue in Figure 1), where the leader can influence the rest of the swarm. The incorporation of the SSA with the CRNN is achieved by formulating the hyperparameters and structure of the latter as an optimization problem. This section, Section III, (A) describes the CRNN, the solution representation is described in Section III (B), and the hybridized SSA is described in Section III (C).

### 3.1. Convolutional Recurrent Neural Network (CRNN)

The convolutional recurrent neural network model was first proposed by [64] to recognize sense text and it outperforms the standard CNN due to end-to-end training using sequence labeling of image words instead of annotation for each character image. Another beneficial aspect of the CRNN for handwriting recognition is its ability to automatically extract valuable features without using traditional handcrafted features.

**Figure 2:**
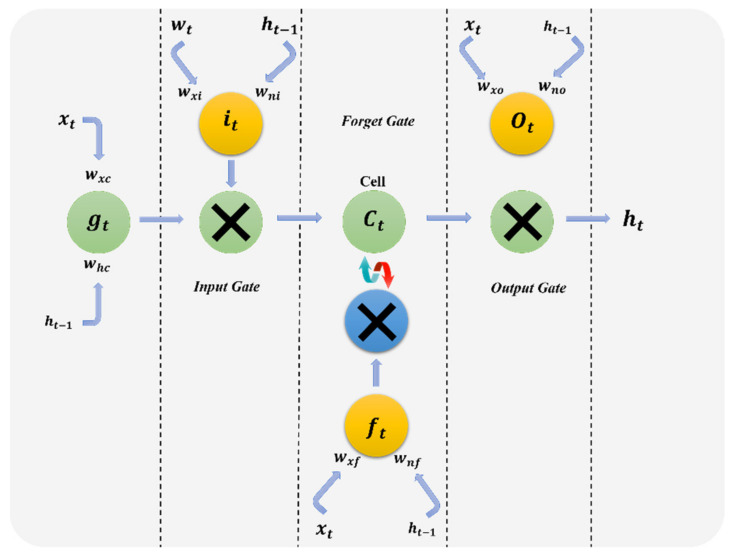
The standard LSTM cell.

In the CRNN model, there is no need to employ a segmentation phase due to the existence of a recurrent layer, which provides high flexibility for handwritten image data, one type of sequence data. The first component in the CRNN model is a convolutional neural network model that includes multiple convolutional layers. Each convolutional layer may contain hyperparameters such as the activation function, batch normalization, the pooling operation, the number of kernels and kernel size, and skip connections. In the CRNN, the convolutional neural network is usually used for feature extraction. The pixel-intensity values are fed to the first convolutional layer and move successively through the CNN. The CNN’s output is a set of feature sequences that are fed to a recurrent neural network (RNN). Finally, the transcription layer, called the connectionist temporal classification (CTC), translates the resulting prediction into a label sequence. In this study, we investigated variants of the RNN known as long short-term memory (LSTM) and bidirectional long short-term memory (BLSTM). The LSTM network structure is based on a gate mechanism capable of solving long-term dependence problems. The gate mechanism includes an input gate, forget gate and output gate. Figure 2 presents the LSTM gate mechanisms’ procedure.

LSTM is often used to model data containing sequence information, such as text and speech, and has been widely used in image processing and natural language processing. The formal definition of LSTM is as follows:

(1) $$i_{t}=\sigma \left(W_{i}\left[x_{t};r_{t-1}\right]+b_{i}\right)$$

(2) $$f_{t}=\sigma \left(W_{f}\left[x_{t};r_{t-1}\right]+b_{f}\right)$$

(3) $$o_{t}=\sigma \left(W_{o}\left[x_{t};r_{t-1}\right]+b_{o}\right)$$

(4) $$c_{t}=i_{t}⊙tanh\left(W_{c}\left[x_{t}\right]+b_{c}\right)+f_{t}⊙c_{t-1}$$

(5) $$h_{t}=o_{t}⊙tanh\left(c_{t}\right)$$

where σ represents the sigmoid function, ⊙ represents bitwise multiplication, and $i_{t}$, $f_{t}$, $o_{t}$ and $c_{t}$ represent the input gate, forget gate, output gate, and cell state of the LSTM at time *t*, respectively. The process of choosing all of the hyperparameter values is explained in detail in the next section. In addition, since there is a recurrent layer in the CRNN model, which provides high flexibility for handwritten image sequence data, there is no need for a segmentation phase.

This study focuses on determining the best CRNN structure and hyperparameters. The next section describes the parameters that need to be optimized.

### 3.2. Solution Representation

In this study, the solution includes three primary components that determine the final CRNN configuration: the general hyperparameters, the convolution hyperparameters, and the long short-term memory network hyperparameters. The general hyperparameters are responsible for providing the hyperparameters for the whole CRNN model as well as the configuration network, which include the batch size, optimizer, learning rate, number of convolution layers, and number of LSTM layers. These hyperparameters are described as follows.

- Batch size: The total number of training samples presented in a single batch, B ∈ (16, 32, 64, 128).
- Optimizer: The adaptive learning rate optimization algorithm is used to iteratively update the CRNN network weights based on the training data. The optimization algorithm can be any of the following: Adam, Nadam, RMSprop, Adadelta, SGD, Adagrad, Adamax.
- Learning rate: The change in the weights during training. The learning rate is represented by one decision variable in the solution, and is one of seven values: LR ∈ (1 × 10^−5^, 5 × 10^−5^, 1 × 10^−4^, 5 × 10^−4^, 1 × 10^−3^, 5 × 10^−3^, 1 × 10^−2^, 1 × 10^−2^, 5 × 10^−2^).
- Number of convolution layers: This decision variable determines how many convolution layers to add to our CRNN. Since handwriting recognition is considered a complex classification task, the first three layers are compulsory in all of the generated individuals (i.e., the first three convolution layers are combined as a fixed layer) to guarantee the automatic detection of the important features. However, an increase in the number of convolution layers can result in an increase in the number of weights as well as the model complexity. Consequently, we limit the convolution layers in our CRNN to 10, and that maximum number of layers can be chosen in our DC-CRNN’s search space.
- Number of LSTM layers: This decision variable determines how many LSTM layers to add to our CRNN.
- Other decision variables used to determine the remaining hyperparameters, which may vary for each convolution layer in the network, are:
- Convolution kernels (ck): The number of kernels in each convolution layer, where ck ∈ (4, 8, 16, 32, 64, 128, 265, 512).
- Convolution kernel size (cs): The kernel size used in each convolution layer, where cs ∈ (2, 3, 4, 5, 6, 7, 8, 9).
- Convolution batch normalization (cb): The use of batch normalization, which is typically utilized to enhance a neural network’s speed and performance. It is applied between the convolution layer and the nonlinearity layers, such as max pooling and ReLU. In our solution, the decision variable for batch normalization is in a binary (0, 1) range.
- Convolution activation function (ca): The usual ReLU is the default and most common activation function used in deep learning networks, especially convolutional neural networks. However, we attempt to choose a more suitable function for our network, which may be: ‘relu’, ‘linear’, ‘elu’, ‘selu’ or ‘tanh’.
- Convolution pooling size (cp): The pooling layer used to reduce the representation size of the input handwritten image, which leads to a reduction in the number of parameters and amount of computation in the network. While the use of pooling layers is important for maintaining a reasonable computation time during the optimization process that finds the optimum network structure, the overuse of pooling layers often removes important features or even reaches a representation size of (1, 1). In our decision variables, we limit the probability of using the pooling after each convolution layer to 50%, the pooling size to (2, 2) and the stride to ∈ {(2, 2), (2, 1)}.
- Skip connection (cr): The use of skip connections, which improve the convergence and performance during training.

Additional decision variables used to determine the hyperparameters for each LSTM layer include the size of the hidden layer (Rh) and the type of LSTM layer, which could be a bidirectional (Rb) layer.

All of these hyperparameters are translated to configure a CRNN structure that runs on part of the dataset to rank the data based on the obtained character error rate (CER), which must be minimized. The solution is optimized using the proposed HSSA and based on the given configuration in Table 1.

### 3.3. Hybrid SSA (HSSA)

The Salp Swarm Algorithm (SSA) is a recent metaheuristic method proposed by [65]. The SSA is inspired by the swarming behavior of salp chains. Salps descend from the Salpidae family and exhibit a transparent, barrel-shaped body. These creatures form a chain to achieve better locomotion using quick, coordinated alterations and foraging. Salp chaining behavior has been mathematically modeled to solve optimization problems [65]. The initial modeling step divides the salp population into two categories: leaders and followers. The leader heads the salp chain, and the rest are considered the followers. Typically, swarm optimization methods simulate individuals by defining their position in an *n*-dimensional searching space, where n is the number of variables in a processed problem. Similarly, the SSA assigns each salp an *n*-dimensional position that is iteratively updated. Therefore, the chain positions are stored in a two-dimensional matrix. Additionally, it is assumed that there is a food source in the search space that is targeted by the storm and denoted by F. In the SSA, the position of the leader is updated as follows:

(6) $$x_{j}^{1}=\left\{\begin{array}{c} F_{j}+c_{1}\left(\left(ub_{j}-lb_{j}\right)c_{2}+lb_{j}\right)\;c_{3}\geq 0\\ F_{j}-c_{1}\left(\left(ub_{j}-lb_{j}\right)c_{2}+lb_{j}\right)\;c_{3}<0 \end{array}\right.$$

where $x_{j}^{1}$ is the *j*th dimension of a chain leader, $F_{j}$ represents the *j*th dimension of the food source, $ub_{j}$ and $lb_{j}$ are the upper and lower boundaries of the *j*th dimension, respectively, and $c_{1}$, $c_{2}$, and $c_{3}$ are random numbers.

Coefficient $c_{1}$ is vital in this equation, as it is responsible for achieving a balance between exploration and exploitation, which is defined as follows:

(7) $$c_{1}=2e^{-{\left(4l/L\right)}^{2}}$$

where  l is the current cycle, and *L* is the maximum number of cycles.

The other two parameters, $c_{2}$ and $c_{3}$, are random numbers generated uniformly in the interval [0, 1]. These parameters dictate whether the next position in the *j*th dimension moves toward negative infinity or positive infinity. Additionally, they determine the step size.

Equation (8) presents the method for updating the leader’s position with respect to the food source. Followers are updated based on Newton’s law of motion as follows:

(8) $$x_{j}^{i}=\frac{1}{2}at^{2}+v_{0}t$$

where i  is the index of the current follower (i>1), $x_{j}^{i}$ is the *j*th dimension of the *i*th follower, *t* is time, *v*_0 is the initial speed, and $a=\frac{v_{final}}{v_{0}}$.

In optimization, time indicates the number of iterations, where the difference between two iterations is one. Thus, by considering that the initial speed is zero (v_0=0), Equation (4) can be expressed as follows:

(9) $$x_{j}^{i}=\frac{1}{2}\left(x_{j}^{i}+x_{j}^{\left(i-1\right)}\right)$$

where i>1, and $x_{j}^{i}$ is the *j*th dimension of the ith follower. All of the SSA steps, which include population initialization and salp movement, are presented in Algorithm 1.
**Algorithm 1:** Salp Swarm Algorithm (SSA)*N → number of salps in the swarm.* *D → number of dimensions of the given problem.* *X → Initialize a swarm of salps with respect to lb and ub.* *F → The best search agent (Food source).* **while** (*Stopping criterion is not met*) **do**       Calculate the fitness of the salps      c_1_ = 2 *x* e**^(*4l=L*)^**      **for** *i = (1 to N)* **do**          **for** *j = (1 to D)*
**do**             **if**
*i = 1* **then**                 Update the position of salps’ leader using Equation (6).              **else** $\;\;\;\;\;\;\;\;\;\;\;\;x_{j}^{i}=\frac{1}{2}\left(x_{j}^{i}+x_{j}^{\left(i-1\right)}\right)\;\;\;$…… ***(See Equation (9))***    **for**
*i = (1 to N)*
**do**       Fit *x^i^* to its boundaries.       **if** *f(x^i^) < f(F)* **then**         F = xi **Output**: F

The SSA uses a set of solutions to find the best possible structure and hyperparameters for the proposed RCNN. That is, the SSA has the ability to explore the problem’s search space. However, it is not as adept at exploitation. Therefore, this study incorporates a local search algorithm for this purpose, as shown in Figure 1. To avoid being trapped in local optima, this study investigates local search methods with acceptance criteria, namely, simulated annealing (SA) and Late Acceptance Hill-Climbing (LAHC). Local search enables the algorithm to identify non-improving solutions so that local optima are ignored.

Generally, this searching process is based on the SSA; using a predefined local search probability (lp), the SSA invokes the local search, which may be LAHC or SA. In this step, the SSA selects the best follower among the salps and passes it to the local search to intensify the search process around its neighborhood, as shown in Algorithm 2.
**Algorithm 2:** Hybridized SSA.**Input:***Handwritten text dataset (sequence of letters and digits)* *N → number of salps in the swarm.* *D → number of dimensions of the given problem.* *X → Initialize a swarm of salps with respect to lb and ub.* *F → The best search agent (Food source).*       **while** (Stopping criterion is not met) **do**            **for each** salp 2 X **do**               Decodes the salp to a CRNN network (*See Section 3.2*)               Train the CRNN on part of the training set.               Evaluates the salp’s fitness based on part of the validation set.               Update the positions of the salps.               *F* Get the best salp.               **for**
*i =(1 to N)* **do**               *Fit xi to its boundaries.*               **if** *f(xi) < f(F)* **then**               *F = xi*               **if**
*rand() < lp* **then**               *F local search(F)* **Output:** *Best CRNN con_guration (F)*

LAHC is a hill-climbing (HC) algorithm with the ability to identify a non-improving solution for the purpose of skipping potential local optima. However, the accepted solution will ideally be of better quality than a solution from previous iterations. That is, the LAHC search procedure depends on the search history, and thus, it maintains a special list for recording that history. In practice, a new solution is compared to the last element in the list. The new solution is chosen as a new searching point if it is better than the rescored value. Then, the quality of the new solution is inserted at the front of the list, and the last element is dropped. These steps are further explained in Algorithm 3.

**Algorithm 3:** Late Acceptance Hill-Climbing (LAHC)X → Initial CRNN structure obtained from the SSA L → length of the list **for** i = 1 to L **do**       fi = f(X).   > Initialize the fitness list. X*= X.             >Memorize the best solution. **for** i = 1 to Max_iterations **do**       X’ = NS(X).    >Move from the current solution to a new one.      v = i mod L      **if** f(X’) ≤ fv ll f(X’) ≤ f(X) **then**             X = X0. >Accept the new solution.             **if** X0 < X_ **then**                   X_ = X0             fv = f(X).     >Insert the current cost to the fitness list. **Output**: X_

The behavior of memorizing the previous iterations is similar to the tabu search (TS) [66]. However, the TS memorizes only the movements, while LAHC memorizes the fitness of previous solutions. Additionally, the TS compares the entire list to the movement that led to a new solution, while LAHC considers only one value from its list. That is, a time complexity of O(1) is required for comparing a new solution to the LAHC list. LAHC accepts non-improving solutions intermediately and memorizes the best solution, which is output by the searching procedure.

SA mimics a physical phenomenon: metal annealing [67]. This activity consists of annealing a metal to obtain a desired shape. In practice, the annealing process includes heating and gradual cooling procedures. Thus, a metal undergoing this process can be shaped as long as a certain temperature is maintained. Throughout the cooling phase, the metal will be gradually hardened, and thus, its shape will become more difficult to adjust. Inspired by this process, SA starts with an initial solution (metal) at a predefined temperature; then, it adjusts the solution until the desired solution is obtained. Accordingly, SA allows less optimal movements for the sake of skipping local optima. However, accepting a less optimal movement is conditioned by Equation (10).

(10) $$e^{\frac{-\Delta E}{T}>R}$$

where *T* is the system temperature, ΔE is the energy difference between the new and current solutions, and *R* is a uniform random number in the interval (0,1).

During the SA process, the temperature is decreased gradually to limit the acceptance of low-quality solutions at late stages in the searching process. In practice, this decrement is implemented using a geometrical scheduler. This scheduler consists of multiplying the current temperature by a cooling factor α, i.e., T=T×α. In this study, the value of α was set to 0.98. To illustrate SA’s algorithmic steps, the pseudocode is given in Algorithm 4.
**Algorithm 4:** Simulated annealing (SA)*X → Initial CRNN structure obtained from the SSA* *T → Initial temperature* *α → Cooling scheduler* *T_f_ → final temperature* X* = X > memorize the best solution         **while**
*(T > T_f_)*
**do**         X’ = NS(X)               **if**
*f(X’)* ≤ *f(X)*
**then**               *X = X’*                     **if**
X’ < X*
**then**                         X* = X’         **else**         **if** $e^{\left(-\Delta E/T\right)\;}>R$   **then**         *X = X’*         T=T*α **Output**: X*

Algorithm 4 shows how the SSA solution (*X*) is improved based on the SA algorithm. SA stops once a final temperature is reached, which is set experimentally in this study to 10^−5^. A new solution is derived from *X* based on a predefined neighborhood structure (NS). NS randomly swaps selected variables from the current solution to obtain a new one. Then, the new solution is evaluated using the fitness function. Typically, the new solution is accepted if its fitness value is better than the current one. Otherwise, the SA acceptance criterion (Equation (10)) is applied to determine whether to accept or reject the new solution.

## 4. Experimental Design

This section presents the experiments conducted in this study. This design includes the experimental setup, data sets, evaluation metrics, and the obtained results. The primary aim of this study is to find the best possible CRNN hyperparameters and structure for the chosen problem. To achieve this goal, several experiments were conducted for this work. These include experiments regarding the encoding schemes used in the optimization process. In this study, direct and indirect encoding are implemented using a standard genetic algorithm. This experiment serves as a baseline for our proposed hybrid SSA. Subsequent experiments are conducted to show the effectiveness of the proposed local search algorithms, i.e., SA and LAHC. Finally, we discuss our proposed method in comparison to the related works.

### 4.1. Implementation Details

The deep neural network training process takes a long time to produce reliable and competitive results. When the training method is used as part of the fitness computation in a swarm optimization algorithm, it significantly increases the processing time. That is, a swarm-based CRNN method that involves a population of 20 individuals evolving for 100 iterations will take 25 days, which is impractical. To address this problem, we trained the network with only 10 epochs in each fitness computation and used a random 25% sample of the training set in each epoch. As a result, we reduced the time requirement of the swarm algorithm to 40 h. To execute these experiments, we used PyTorch [68], an open-source machine learning library on a cluster of NVIDIA 2080-Ti GPUs with a processer of 4.3 GHz, the AMD Ryzen7 2700x. The hyperparameter values have been tuned based on the proposed swarm algorithm as described in Section 3.2.

### 4.2. Dataset

The proposed DC-CRNN is tested on two distinct languages: English and Arabic. The English language dataset is called the IAM dataset, and the Arabic language dataset is called the IFN/ENIT dataset. Due to the importance of the training-testing ratio of each dataset, we used the official training, testing quantities which is about 80% for training and 20% for testing have been published for both (Arabic and English) datasets. This makes it easier to compare with other works. This section presents the details of these data sets.

#### 4.2.1. English Sequence Handwriting Dataset

We used the English dataset, called IAM, which is widely used in the field of document analysis. The IAM dataset comprises 115,320 segmented and transcribed word images of handwritten text. English texts from 657 writers were collected for the data set. The dataset includes forms of unconstrained handwriting text, which were scanned at a resolution of 300 dpi and saved as PNG images with 256 gray levels. One advantage of the data set is that official training, testing and validation quantities have been published. This makes it easier to compare with other work.

#### 4.2.2. Arabic Sequence Handwriting Dataset

An Arabic dataset, called the Institute of Communications Technology/Ecole Nationale d’Ingénieurs de Tunis (IFN/ENIT) dataset [69,70], was used in this research to demonstrate the impact of using different languages on our model’s performance. IFN/ENIT contains 32,492 handwritten word images, and each word presents a name of a Tunisian city or village. The dataset includes contributions from over four hundred writers so that it includes several varieties of writing styles. The dataset was separated into five sets: A, B, C, D, and E. The first four sets were adopted for training, while set E was used for testing. The training sets include a total of 26,459 images, with 6033 images for testing.

### 4.3. Evaluation Metrics

The system performance was evaluated using the Word Error Rate (WER) and Character Error Rate (CER) metrics. The CER expression is specified below,

(11) $$\mathrm{CER}=\frac{S+I+D}{N}\;\;\;\;\;$$

where *S*, *D*, and *I* denote the character count substitutions, deletions, and insertions, respectively. *N* denotes the total number of characters that compose the original transcription.

(12) $$\mathrm{WER}=\frac{Sw+Iw+Dw}{Nw}$$

The WER calculation is similar to that of the CER. Here, *Sw*, *Iw*, *Dw*, and *Nw* represent words. The following experiments constrain the WER and CER to a range of 0–100. Here, lower values indicate higher performance.

### 4.4. Convergence Analysis of the Proposed Method

**Figure 3:**
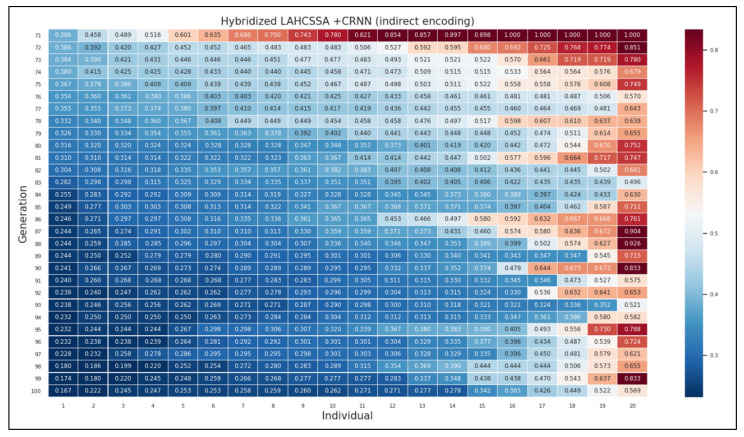
The convergence behavior of the hybrid LAHCSSA when optimizing the CRNN.

This section discusses the convergence behavior of the hybrid SSA when it is used to optimize the CRNN. In terms of convergence, we present the results of implementing neuroevolution using the genetic algorithm as an optimization method. We implemented the genetic algorithm for comparison purposes due to the lack of works that implemented the evolution-based CRNN for the sequence modeling task of handwriting recognition. In this implementation, we examined two encoding schemes: direct and indirect encoding. We included both to measure their impact on the genetic algorithm when it is used to optimize the CRNN. The second experiment shows the effectiveness of the proposed SSA with respect to the encoding schemes. Additionally, the results of the hybridized SSA with LAHC and SA are presented with respect to these encoding schemes (see Appendix A). The fitness of each individual is displayed to show the proposed algorithm’s convergence (see Figure 3). 

**Figure 4:**
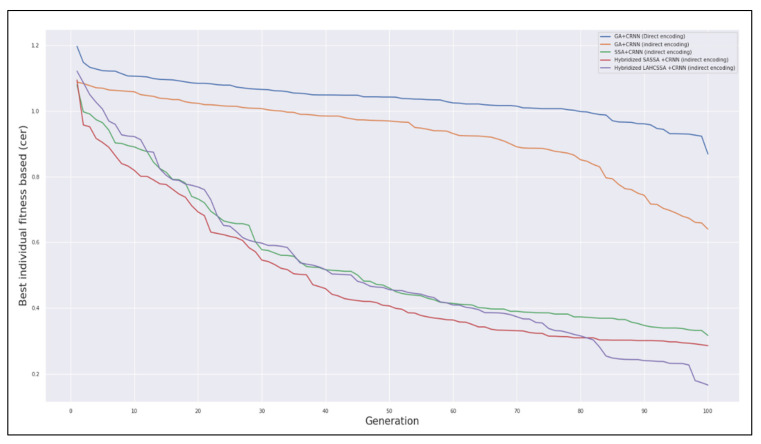
Best individual fitness from each optimization technique.

Figure 3 shows the obtained CER over the last thirty generations for 20 individuals. For each individual, a corresponding CRNN is built, and the CER is then calculated. The individual with the minimum CER is the best solution obtained by the GA or the hybrid SSA. For ease of interpretation, the heat map in Figure 3 uses color as a reference, where brown refers to a high CER (low-quality solutions) and navy refers to a low CER (high-quality solutions). Based on this color coding, navy individuals are increased in the last generations. That is, the algorithm converges toward high-quality solutions. Similarly, the number of brown individuals is significantly decreased, as only the fittest individuals survive throughout the generations. To demonstrate the effectiveness of the proposed hybrid SSA, we compare it against the genetic algorithm, as shown in Figure 4.

**Figure 5:**
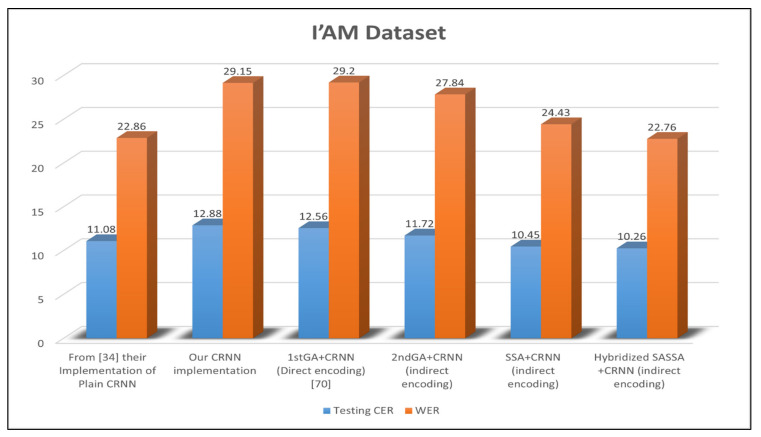
Results of the CRNN variants based on the IAM Dataset. Bold values indicate the best results for the corresponding experimental configurations.

Figure 4 shows the fitness of the best individual across all optimization techniques. These techniques include the genetic algorithm, the SSA hybridized with SA, and the SSA hybridized with LAHC. This comparison indicates that the SSA performs better than the genetic algorithm; this result is attributed to the SSA swarm behavior, as it converges toward better optima faster than the GA. Additionally, the SSA outperformed other techniques when it is hybridized with LAHC, as LAHC enforces the SSA exploitation process. Hybridizing the SSA with LAHC revealed better exploitation than using SA as local search. The latter requires a lengthy annealing process to obtain the desired CRNN structure and hyperparameters. Figure 5 reveals that the CRNN structure output by the hybrid SSA is the largest among the methods. This is due to the algorithm’s exploitation process, which was enforced using simulated annealing (SA). This result can be clearly seen when it is compared to the original SSA, where the latter produced a smaller network by 0.5 million parameters than the hybrid SSA. That is, the hybrid SSA makes a tradeoff between the network size and accuracy. Therefore, the hybrid SSA was able to obtain the best accuracy in comparison to other methods.

### 4.5. Ablation Experiment from the Optimized CRNN

To show the impact of the optimization model on the CRNN An ablation study we removed all certain components like pre/post-processing to highlight ablation experiment results. we compared three types of optimization methods against each other. These methods are the GA, SSA, and Hybridized SSA. We first tested our system on the IAM English handwritten dataset. Figure 5 shows that the CRNN-based hybrid SSA is superior to the other models. Additionally, this comparison includes the handcrafted CRNN as a baseline model, which has been implemented in this study others [36].

The handcrafted CRNN used in this study is slightly inferior to the one in [36], which can be attributed to the parameter settings. To show the impact of the solution’s encoding, we examined direct and indirect encoding with the GA, i.e., GA1 and GA2. Indirect encoding may minimize the error rate to 11.72% for the CER and 27.84% for the WER, which outperforms direct encoding. Thus, only indirect encoding is used for the SAA and hybrid SSA (SSA with SA and SSA with LAHC). The CRNN-based SSA obtained an improved CER and WER in comparison to GA1 and GA2. Similarly, the CRNN-based hybrid SSA achieved a minimal CER and WER in comparison to the other models. The effectiveness of the CRNN-based hybrid SSA is attributed to the swarm behavior of the SSA and its improved local search process. The local search in turn is tested using the LAHC and SA algorithms. The SSA achieves better performance when it is hybridized with LAHC rather than SA.

**Figure 6:**
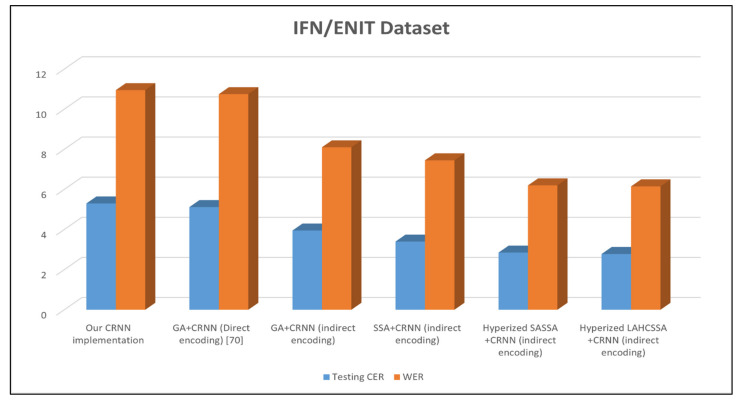
Results of the CRNN variants using the IFN/ENIT Dataset. Bold values indicate the best results for the corresponding experimental configurations.

**Figure 7:**
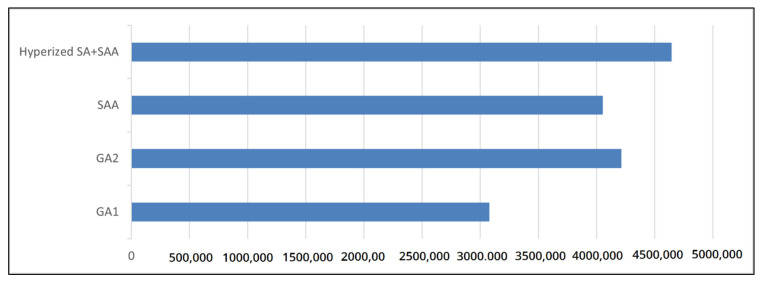
The number of parameters for each network.

Additionally, the CRNN-based hybrid SSA outperformed the other models when they were tested on IFN/ENIT, as shown in Figure 6. In Figure 6, the baseline model achieved 5.3% and 10.95% for the CER and WER, respectively. The CRNN-based hybrid SSA could improve this result by approximately 50% for both the CER and WER. However, the CRNN-based GA1 was slightly better than the baseline model. Additionally, the CRNN-based GA2 and SSA were able to minimize the CER and WER by approximately 30%. Based on these results, the hybridized SSA with LAHC was the best model for optimizing the CRNN-based model. However, this model yielded a larger network than other models, as shown in Figure 7.

### 4.6. Reliability of the Proposed DC-CRNN

The proposed DC-CRNN (a CRNN optimized by the SSA hybridized with LAHC) has shown an efficient and consistent performance in comparison to the other models implemented in this study (see Section 4.5). However, a comparison to related sequential handwriting models is required to prove the reliability of the DC-CRNN. In this study, we compared the DC-CRNN to eight state-of-the-art algorithms applied to the IAM dataset based on the word-level and without pre-trained models, as shown in Table 2. Ref. [32] included an SVM with the CNN for the recognition task, and it also included a postprocessing step based on a lexicon approach. This work limits the transcription to words appearing in lexicons derived from the IAM test set. The second method is based on the CRNN with a preprocessing step that includes simple slant correction and segments lines into words using vertical and horizontal projections. Additionally, the CRNN in [33] was followed by a language model called WFST. The method in [34] is based on the CNN-FCN, which uses a pretrained model and probabilistic CER correction based on a lexicon. 

Ref. [35] proposed a CRNN-STN based on a pretrained model using synthetic data. Additionally, their model was tested with and without a lexicon as a postprocessing step. Ref. [36] developed a CRNN-STN with preprocessed data using slant and slope normalization, as well as postprocessing based on a test set lexicon. Additionally, the CRNN-STN was trained on a pretrained model and three types of augmentation schemes with test time augmentation. Ref. [37] binarized the IAM dataset before using the CRNN. Ref. [40] used sequence-to-sequence (Seq2Seq) preceded by an augmentation step, and this study built their model on a pretrained model. Additionally, [40] implemented a language model named candidate fusion as a postprocessing step. The eighth work used a CRNN proceeded by a language model, yet the study did not include a preprocessing stage.

**Figure 8:**
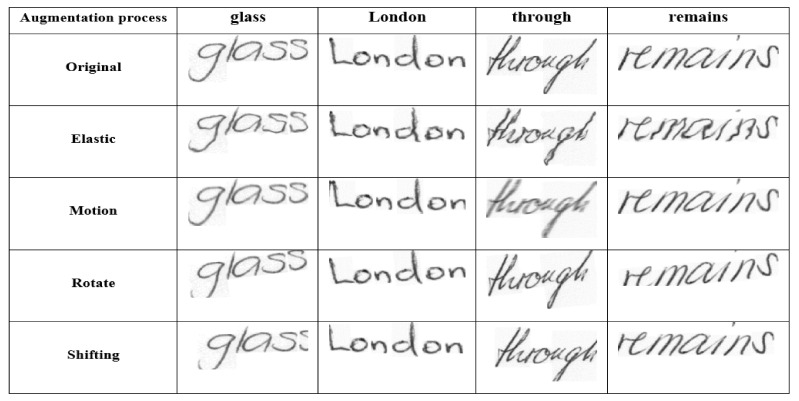
An Augmentation Samples from the IAM dataset.

In comparison to the aforementioned works, our proposed model outperforms all of them in terms of the WER and CER. However, the model in [37] reported a slightly smaller WER than our model due to their usage of a different testing set called (Parzival dataset). Similarly, some works in Table 2 imported a pretrained model that was intensively trained. Our model was trained from scratch and was able to outperform many of the previous works. However, to ensure appropriate comparison to the related works, we incorporated an augmentation procedure as a preprocessing step, as shown in Figure 8. Additionally, our DC-CRNN is followed by WER correction based on the lexicon as a postprocessing step.

In addition to the reported results in Table 2, there are studies that obtained CER of 2.52% [36], and 3.2% [20] for the IAM dataset. In [20], the authors addressed the problem based on sentences which made it easier to scan the words with the help of NLP prediction tools. In comparison to our proposed method, these methods have obtained lower CER for the IAM dataset. However, our method was able to scale well for other datasets of different languages, i.e., the Arabic dataset, which carries entirely different features. That is, our method does not rely on domain-specific NLP procedures, nor a pre-trained model of a specific language. Considering these factors, our method was able to outperform others when word-level is addressed. However, our method is not the best for OCR in comparison to the related works. 

Additionally, the proposed method was compared to related works using the IFN/ENIT dataset (Table 3). This comparison includes seven distinct methods. The first [71] is based on a dynamic temporal residual network for sequence modeling, (DTRN), which obtained a 6.91% CER, while the WER was not reported. Ref. [72] achieved a 12.6% WER using one-dimensional LSTM. A multidimensional LSTM was used by [73], which achieved an 11.62% CER. The method in [74] is based on a convolutional deep belief network (CDBN) and obtained a 7.1% WER. Ref. [75] obtained a 4.8% WER using the CBN and CNN. Ref. [76] proposed multidimensional LSTM, which achieved a 10.11% WER. Ref. [77] is based on bi-LSTM (BLSTM) and recorded a 4.5% WER.

Most of the mentioned methods reported the WER due to the original dataset labels, which only include words.

In comparison to the aforementioned methods, our method was able to minimize the CER and WER to 2.20% and 4.45%, respectively. These results show a more than 50% improvement over 1D LSTM due to the two-dimensional LSTM included in the proposed CRNN. Similarly, an approximate 50% improvement was obtained when compared to [76] as shown in Table 3. Additionally, the proposed method was better than the other methods by at least 5% when compared to the BLSTM. This result is mainly attributed to the automatic configuration of the proposed CRNN, which is achieved based on the hybrid SSA. The hybrid SSA enabled the proposed model to obtain the structure and hyperparameters that suit the IFN/ENIT dataset.

## 5. Conclusions and Future Work

This study presented a dynamically configurable CRNN (DC-CRNN) for text sequence modeling. The CRNN’s dynamic configuration was achieved using an SSA hybridized with LAHC. That is, the best CRNN structure and hyperparameters are formulated as an optimization problem, where the SSA is applied to solve it. The SSA algorithm is based on the notion of swarm optimization, and thus, it emphasizes exploring the problem space rather than exploiting it. To balance the search process, we hybridized the SSA with a local search to enhance its exploitation capacity. The optimal choice for the local search was investigated using the SA and LAHC algorithms. LAHC has demonstrated better performance than SA. The DC-CRNN showed significant improvement over CRNNs with handcrafted configurations. Additionally, we implemented the DC-CRNN based on the genetic algorithm for comparison purposes. Note that optimizing the CRNN using a metaheuristic process is expensive in terms of the runtime. Therefore, when we optimized the CRNN, we shrunk the training data and used fewer epochs. Our model has obtained 3.40% and 6.18% for CER and WER, respectively. These results were the best in comparison to related CRNN works when word-level is considered and without using pre-trained models. In future research, other metaheuristics can be investigated to optimize the CRNN.

## Figures and Tables

**Table 1:** CRNN configurations solution representation.

Decision Variable Sectors	Total Decision Variable Bits for Each Sector	Hyperparameters	No. of Bits for Each Hyperparameter
General parameters	10 bits	Bs (batch size)	2
		Op (optimizer)	1
		Lr (learning rate)	3
		Nc (number of convolution layers)	2
		Nr (number of LSTM layers)	2
Convolution layer parameters × 7	11 bits × 7 layers = 77 bits	Ck (number of kernels)	3
		Cs (kernel size)	3
		Cb (batch normalization)	1
		Ca (activation function)	1
		Cp (pooling size)	2
		Cr (skip connection or not)	1
Recurrent network parameters × 4	3 bits × 4 layers = 12 bits	Rh (size of hidden layer)	2
		Rb (bidirectional)	1

**Table 2:** A comparison to CRNN related works using the IAM Dataset without a pre-trained model. Bold values indicate the best results for the corresponding experimental configurations.

No.	Recognizer Model	CER	WER
1.	[32]	3.72	6.69
2.	[33]	4.80	9.30
3.	[34]	4.70	8.22
4.	[35]	6.34	16.19
		3.50	9.30
5.	[36]	11.08	22.86
		4.88	12.61
6.	[37]	3.30 Parzival	No
7.	[40]	4.27	8.36
8.	[41]	8.5	--
9.	Proposed method	3.40	6.18

**Table 3:** A comparison against the related works based on the IFN/ENIT dataset.

No.	Recognizer Model	CER	WER
1.	[71]	6.91	--
2.	[72]	--	12.60
3.	[73]	11.62	--
4.	[74]	--	7.10
5.	[75]	--	4.80
6.	[76]	--	10.11
7.	[77]	--	4.50
8.	Proposed method	2.20	4.45

**Figure A1:**
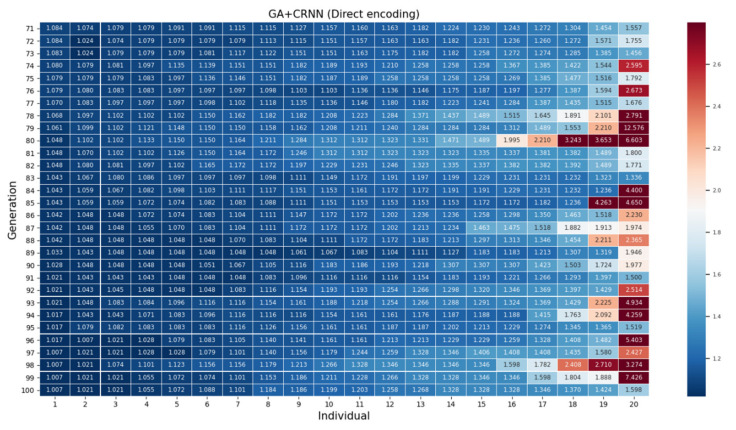
GACRNN direct encoding.

**Figure A2:**
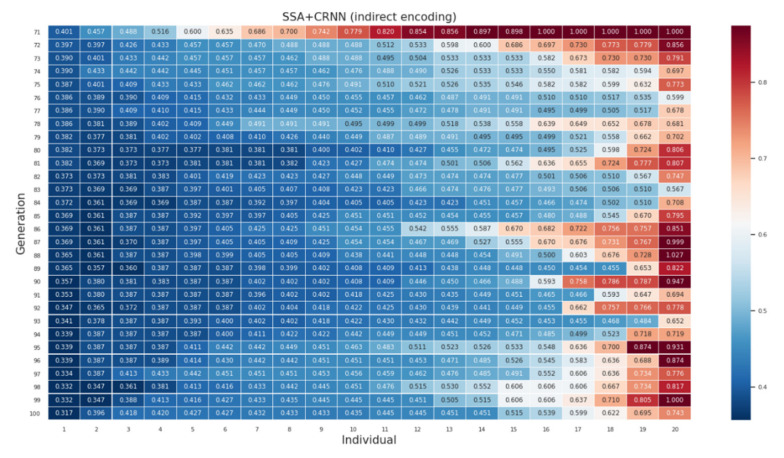
GACRNN indirect encoding.

**Figure A3:**
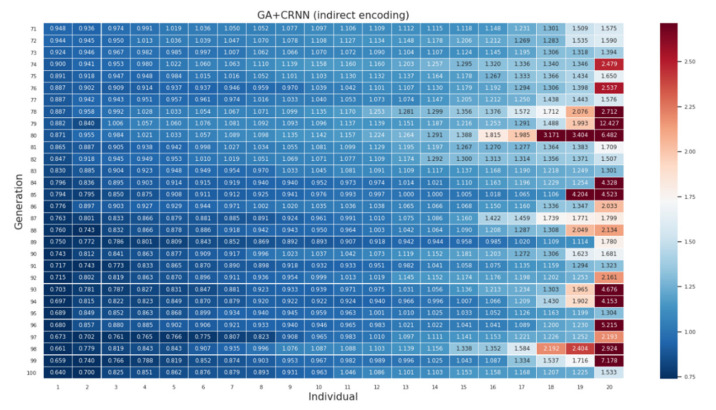
(SSACRNN).

**Figure A4:**
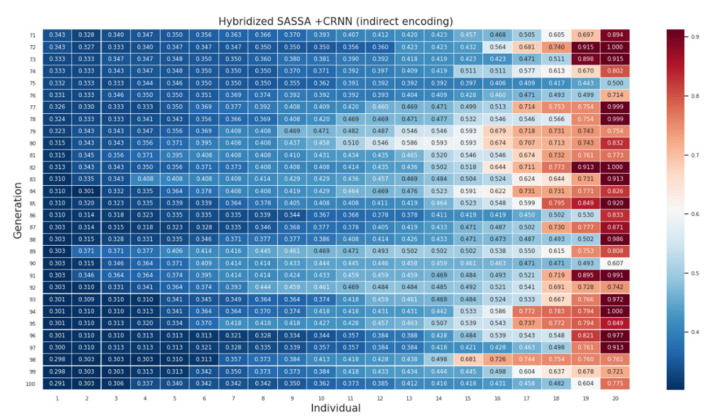
(SASSACRNN).

## Abbreviations

DC-CRNN	Dynamically Configurable Convolutional Recurrent Neural Network
SSA	Salp Swarm Optimization Algorithm
SA	Simulated Annealing
HC	Hill Climbing
LAHC	Late Acceptance Hill-Climbing
TS	Tabu Search
lp	Local Search Probability
RNNs	Recurrent Neural Networks
CRNNs	Convolutional Recurrent Neural Networks
CNNs	Convolutional Neural Networks
NAS	Neural Architecture Search
LSTM	Long Short-Term Memory
WFST	Weighted Finite-State Transducer
CTC	Connectionist Temporal Classification
BLSTM	Bidirectional Long Short-Term Memory
STN	Spatial Transformer Network
TL	Transfer Learning
seq-to-seq	Sequence-To-Sequence
LDN	Language Denoiser Network
NEAT	Neuroevolution Of Augmenting Topologies
PSO	Particle Swarm Optimization
QBPSO	Particle Swarm Optimization With Binary Encoding
ACO	Ant Colony Optimization
HSSA	Hybrid Salp Swarm Optimization Algorithm
IFN/ENIT	Technology/Ecole Nationale d’Ingénieurs De Tunis
WER	Word Error Rate
CER	Character Error Rate

## References

[1] Al-Saiagh W., Tiun S., Al-Saffar A., Awang S., Al-Khaleefa A. (2018). Word sense disambiguation using hybrid swarm intelligence aroach. PLoS ONE.

[2] Zin T.T., Pwint M.Z., Thant S. A Mobile Alication for Offline Handwritten Character Recognition. Proceedings of the 2020 IEEE 9th Global Conference on Consumer Electronics (GCCE).

[3] Hopcan S., Tokel S.T. (2021). Exploring the effectiveness of a mobile writing alication for suorting handwriting acquisition of students with dysgraphia. Educ. Inf. Technol..

[4] Sharma A., Jayagopi D.B. (2021). Towards efficient unconstrained handwriting recognition using Dilated Temporal Convolution Network. Expert Syst. Appl..

[5] Ahmed R., Gogate M., Tahir A., Dashtipour K., Al-Tamimi B., Hawalah A., El-Affendi M.A., Hussain A. (2021). Deep Neural Network-Based Contextual Recognition of Arabic Handwritten Scripts. Entropy.

[6] Gao Y., Xiao G. (2020). Real-time chinese traffic warning signs recognition based on cascade and CNN. J. Real-Time Image Process..

[7] Zhang Y.-J. (2021). Alication of Image Technology. Handbook of Image Engineering.

[8] Hwang S.-M., Yeom H.-G. (2021). An Implementation of a System for Video Translation Using OCR. Software Engineering in IoT, Big Data, Cloud and Mobile Computing.

[9] Zhao B., Tao J., Yang M., Tian Z., Fan C., Bai Y. (2020). Deep imitator: Handwriting calligraphy imitation via deep attention networks. Pattern Recogn..

[10] Awang S., Azmi N.M.A.N., Rahman M.A. (2020). Vehicle type classification using an enhanced sparse-filtered convolutional neural network with layer-skiing strategy. IEEE Access.

[11] Cakic S., Ismailisufi A., Popovic T., Krco S., Gligoric N., Kupresanin S., Maras V. Digital Transformation and Transparency in Wine Suly Chain Using OCR and DLT. Proceedings of the 2021 25th International Conference on Information Technology (IT).

[12] Georgieva P., Zhang P. Optical Character Recognition for Autonomous Stores. Proceedings of the 2020 IEEE 10th International Conference on Intelligent Systems (IS).

[13] Goodfellow I., Bengio Y., Courville A., Bengio Y. (2016). Deep Learning.

[14] Alkhateeb J.H. (2020). An Effective Deep Learning Approach for Improving Off-Line Arabic Handwritten Character Recognition. Int. J. Softw. Eng. Comput. Syst..

[15] Ball G.R., Srihari S.N., Srinivasan H. Segmentation-based and segmentation-free methods for spotting handwritten arabic words. Proceedings of the Tenth International Workshop on Frontiers in Handwriting Recognition.

[16] Biadsy F., Saabni R., El-Sana J. (2011). Segmentation-free online arabic handwriting recognition. Int. J. Pattern Recogn. Artif. Intell..

[17] Rusinol M., Aldavert D., Toledo R., Lladós J. Browsing heterogeneous document collections by a segmentation-free word spotting method. Proceedings of the 2011 International Conference on Document Analysis and Recognition.

[18] Dwivedi A., Saluja R., Sarvadevabhatla R.K. An OCR for Classical Indic Documents Containing Arbitrarily Long Words. Proceedings of the IEEE/CVF Conference on Computer Vision and Pattern Recognition Workshops.

[19] Carbune V., Gonnet P., Deselaers T., Rowley H.A., Daryin A., Calvo M., Wang L.-L., Keysers D., Feuz S., Gervais P. (2020). Fast multi-language LSTM-based online handwriting recognition. Int. J. Doc. Anal. Recogn..

[20] Bluche T., Messina R. Gated convolutional recurrent neural networks for multilingual handwriting recognition. Proceedings of the 2017 14th IAPR International Conference on Document Analysis and Recognition (ICDAR).

[21] Xie Z., Sun Z., Jin L., Feng Z., Zhang S. Fully convolutional recurrent network for handwritten chinese text recognition. Proceedings of the 2016 23rd International Conference on Pattern Recognition (ICPR).

[22] Zhan H., Lyu S., Tu X., Lu Y. (2019). Residual CRNN and Its Alication to Handwritten Digit String Recognition. Proceedings of the International Conference on Neural Information Processing, Sydney, Australia, 12–15 December 2019.

[23] He K., Zhang X., Ren S., Sun J. Deep residual learning for image recognition. Proceedings of the IEEE Conference on Computer Vision and Pattern Recognition.

[24] Mnih V., Kavukcuoglu K., Silver D., Graves A., Antonoglou I., Wierstra D., Riedmiller M. (2013). Playing atari with deep reinforcement learning. arXiv.

[25] Szegedy C., Liu W., Jia Y., Sermanet P., Reed S., Anguelov D., Erhan D., Vanhoucke V., Rabinovich A. Going deeper with convolutions. Proceedings of the IEEE Conference on Computer Vision and Pattern Recognition.

[26] Srivastava N., Hinton G., Krizhevsky A., Sutskever I., Salakhutdinov R. (2014). Dropout: A simple way to prevent neural networks from overfitting. J. Mach. Learn. Res..

[27] Ioffe S., Szegedy C. Batch normalization: Accelerating deep network training by reducing internal covariate shift. Proceedings of the International Conference on Machine Learning.

[28] Yuan W., Dong B., Wang S., Unoki M., Wang W. (2021). Evolving Multi-Resolution Pooling CNN for Monaural Singing Voice Separation. IEEE/ACM Trans. Audio Speech Lang. Process..

[29] Sun D., Wei E., Ma Z., Wu C., Xu S. (2021). Optimized CNNs to Indoor Localization through BLE Sensors Using Improved PSO. Sensors.

[30] Stanovov V., Akhmedova S., Semenkin E. (2021). Neuroevolution of augmented topologies with difference-based mutation. IOP Conference Series: Materials Science and Engineering.

[31] Galván E., Mooney P. (2021). Neuroevolution in deep neural networks: Current trends and future challenges. arXiv.

[32] Krishnan P., Dutta K., Jawahar C. Deep feature embedding for accurate recognition and retrieval of handwritten text. Proceedings of the 2016 15th International Conference on Frontiers in Handwriting Recognition (ICFHR).

[33] Rawls S., Cao H., Kumar S., Natarajan P. Combining convolutional neural networks and lstms for segmentation-free ocr. Proceedings of the 2017 14th IAPR international conference on document analysis and recognition (ICDAR).

[34] Ptucha R., Such F.P., Pillai S., Brockler F., Singh V., Hutkowski P. (2019). Intelligent character recognition using fully convolutional neural networks. Pattern Recogn..

[35] Krishnan P., Dutta K., Jawahar C. Word spotting and recognition using deep embedding. Proceedings of the 2018 13th IAPR International Workshop on Document Analysis Systems (DAS).

[36] Dutta K., Krishnan P., Mathew M., Jawahar C. Improving cnn-rnn hybrid networks for handwriting recognition. Proceedings of the 2018 16th International Conference on Frontiers in Handwriting Recognition (ICFHR).

[37] Jaramillo J.C.A., Murillo-Fuentes J.J., Olmos P.M. Boosting handwriting text recognition in small databases with transfer learning. Proceedings of the 2018 16th International Conference on Frontiers in Handwriting Recognition (ICFHR).

[38] Marti U.-V., Bunke H. (2002). The IAM-database: An English sentence database for offline handwriting recognition. Int. J. Doc. Anal. Recognit..

[39] Fischer A., Keller A., Frinken V., Bunke H. (2012). Lexicon-free handwritten word spotting using character HMMs. Pattern Recog. Lett..

[40] Kang L., Riba P., Villegas M., Fornés A., Rusiñol M. (2021). Candidate fusion: Integrating language modelling into a sequence-to-sequence handwritten word recognition architecture. Pattern Recogn..

[41] Chung J., Delteil T. A computationally efficient pipeline aroach to full page offline handwritten text recognition. Proceedings of the 2019 International Conference on Document Analysis and Recognition Workshops (ICDARW).

[42] Yao X., Liu Y. (1997). A new evolutionary system for evolving artificial neural networks. IEEE Trans. Neural Netw..

[43] Stanley K.O., Miikkulainen R. (2002). Evolving neural networks through augmenting topologies. Evol. Comput..

[44] Kassahun Y., Sommer G. Efficient reinforcement learning through Evolutionary Acquisition of Neural Topologies. Proceedings of the European Symposium On Artificial Neural Networks, Computational Intelligence and Machine Learning, ESANN.

[45] Stanley K.O., Clune J., Lehman J., Miikkulainen R. (2019). Designing neural networks through neuroevolution. Nat. Mach. Intell..

[46] Liu H., Simonyan K., Vinyals O., Fernando C., Kavukcuoglu K. (2017). Hierarchical representations for efficient architecture search. arXiv.

[47] Real E., Moore S., Selle A., Saxena S., Suematsu Y.L., Tan J., Le Q.V., Kurakin A. Large-scale evolution of image classifiers. Proceedings of the International Conference on Machine Learning, PMLR.

[48] Sun Y., Xue B., Zhang M., Yen G.G., Lv J. (2020). Automatically designing CNN architectures using the genetic algorithm for image classification. IEEE Trans. Cybern..

[49] Talbi E.-G. (2021). Automated Design of Deep Neural Networks: A Survey and Unified Taxonomy. ACM Comput. Surv. (CSUR).

[50] Katona A., Lourenço N., Machado P., Franks D.W., Walker J.A. Utilizing the Untaed Potential of Indirect Encoding for Neural Networks with MetaLearning. Proceedings of the Evostar.

[51] Miikkulainen R., Liang J., Meyerson E., Rawal A., Fink D., Francon O., Raju B., Shahrzad H., Navruzyan A., Duffy N. (2019). Evolving deep neural networks. Artificial Intelligence in the Age of Neural Networks and Brain Computing.

[52] Baldominos A., Saez Y., Isasi P. (2018). Evolutionary convolutional neural networks: An alication to handwriting recognition. Neurocomputing.

[53] Fielding B., Zhang L. (2018). Evolving image classification architectures with enhanced particle swarm optimisation. IEEE Access.

[54] Li Y., Xiao J., Chen Y., Jiao L. (2019). Evolving deep convolutional neural networks by quantum behaved particle swarm optimization with binary encoding for image classification. Neurocomputing.

[55] Tan T.Y., Zhang L., Lim C.P. (2020). Adaptive melanoma diagnosis using evolving clustering, ensemble and deep neural networks. Knowl.-Based Syst..

[56] Rosa G., Papa J., Marana A., Scheirer W., Cox D. (2015). Fine-tuning convolutional neural networks using harmony search. Proceedings of the Iberoamerican Congress on Pattern Recognition.

[57] Khalifa M.H., Ammar M., Ouarda W., Alimi A.M. Particle swarm optimization for deep learning of convolution neural network. Proceedings of the 2017 Sudan Conference on Computer Science and Information Technology (SCCSIT).

[58] Ororbia A., ElSaid A., Desell T. Investigating recurrent neural network memory structures using neuro-evolution. Proceedings of the Genetic and Evolutionary Computation Conference.

[59] Bayer J., Wierstra D., Togelius J., Schmidhuber J. (2009). Evolving memory cell structures for sequence learning. Proceedings of the International Conference on Artificial Neural Networks.

[60] Rawal A., Miikkulainen R. Evolving deep LSTM-based memory networks using an information maximization objective. Proceedings of the Genetic and Evolutionary Computation Conference 2016.

[61] Chandra R., Chand S. (2016). Evaluation of co-evolutionary neural network architectures for time series prediction with mobile alication in finance. Appl. Soft Comput..

[62] Desell T., Clachar S., Higgins J., Wild B. (2015). Evolving deep recurrent neural networks using ant colony optimization. Proceedings of the European Conference on Evolutionary Computation in Combinatorial Optimization.

[63] ElSaid A., Jamiy F.E., Higgins J., Wild B., Desell T. Using ant colony optimization to optimize long short-term memory recurrent neural networks. Proceedings of the Genetic and Evolutionary Computation Conference.

[64] Shi B., Bai X., Yao C. (2016). An end-to-end trainable neural network for image-based sequence recognition and its alication to scene text recognition. IEEE Trans. Pattern Anal. Mach. Intell..

[65] Mirjalili S., Gandomi A.H., Mirjalili S.Z., Saremi S., Faris H., Mirjalili S.M. (2017). Salp Swarm Algorithm: A bio-inspired optimizer for engineering design problems. Adv. Eng. Softw..

[66] Glover F. (1986). Future paths for integer programming and links to artificial intelligence. Comput. Oper. Res..

[67] Kirkpatrick S., Gelatt C.D., Vecchi M.P. (1983). Optimization by simulated annealing. Science.

[68] Ketkar N. (2017). Introduction to pytorch. Deep Learning with Python.

[69] El Abed H., Margner V. The IFN/ENIT-database-a tool to develop Arabic handwriting recognition systems. Proceedings of the 2007 9th International Symposium on Signal Processing and Its Alications.

[70] Pechwitz M., Maddouri S.S., Märgner V., Ellouze N., Amiri H. (2002). IFN/ENIT-database of handwritten Arabic words. Proceedings of the CIFED, Hammamet, Tunisia, 21–23 October 2002.

[71] Yan R., Peng L., Xiao S., Johnson M.T., Wang S. (2019). Dynamic temporal residual network for sequence modeling. Int. J. Doc. Anal. Recogn..

[72] Yousefi M.R., Soheili M.R., Breuel T.M., Stricker D. A comparison of 1D and 2D LSTM architectures for the recognition of handwritten Arabic. Proceedings of the Document Recognition and Retrieval XXII, International Society for Optics and Photonics.

[73] Maalej R., Kherallah M. (2016). Improving MDLSTM for offline Arabic handwriting recognition using dropout at different positions. Proceedings of the International Conference on Artificial Neural Networks, Vancouver, BC, Canada, 24–29 July 2016.

[74] Elleuch M., Kherallah M. (2019). Boosting of deep convolutional architectures for Arabic handwriting recognition. Int. J. Multimed. Data Eng. Manag..

[75] Khémiri A., Echi A.K., Elloumi M. (2019). Bayesian versus convolutional networks for Arabic handwriting recognition. Arab. J. Sci. Eng..

[76] Maalej R., Kherallah M. (2019). Maxout into MDLSTM for offline Arabic handwriting recognition. Proceedings of the International Conference on Neural Information Processing, Sydney, Australia, 12–15 December 2019.

[77] Eltay M., Zidouri A., Ahmad I. (2020). Exploring deep learning aroaches to recognize handwritten arabic texts. IEEE Access.

